# Agreement between patient reported outcomes and clinical reports after radical prostatectomy - a prospective longitudinal study

**DOI:** 10.1186/s12894-019-0467-3

**Published:** 2019-05-08

**Authors:** David Bock, Eva Angenete, Anders Bjartell, Jonas Hugosson, Gunnar Steineck, Sofie Walming, Peter Wiklund, Eva Haglind

**Affiliations:** 1 Department of Surgery, Institute of Clinical Sciences, Sahlgrenska Academy, University of Gothenburg, SSORG (Scandinavian Surgical Outcomes Research Group), Sahlgrenska University Hospital/Östra, 416 85 Gothenburg, Sweden; 2 Health Metrics Unit, Sahlgrenska Academy, University of Gothenburg, Gothenburg, Sweden; 3 Department of Urology, Skåne University Hospital, Malmö, Sweden; 4 Department of Urology, Institute of Clinical Sciences, Sahlgrenska Academy, University of Gothenburg, Sahlgrenska University Hospital, Gothenburg, Sweden; 5 Division of Clinical Cancer Epidemiology, Department of Oncology, Institute of Clinical Sciences, Sahlgrenska Academy, Gothenburg, Sweden; 6 Department of Molecular Medicine and Surgery, Section of Urology, Karolinska Institutet, Stockholm, Sweden

**Keywords:** Agreement, Questionnaire, Prostate cancer, Prostatectomy, Case-report form

## Abstract

**Background:**

In clinical research information can be retrieved through various sources. The aim is to evaluate the agreement between answers in patient questionnaires and clinical reports in a study of patients after radical prostatectomy and patient characteristics associated with agreement between these two data sources.

**Methods:**

In the prospective non-randomized longitudinal trial LAParoscopic Prostatectomy Robot Open (LAPPRO) 4003 patients undergoing radical prostatectomy at 14 centers in Sweden were followed. Analysis of agreement is made using a variety of methods, including the recently proposed Gwet’s AC1, which enables us to handle the limitations of Cohen’s Kappa where agreement depends on the underlying prevalence.

**Results:**

The incidence of postoperative events was consistently reported higher by the patient compared with the clinical reports for all outcomes. Agreement regarding the absence of events (negative agreement) was consistently higher than agreement regarding events (positive agreement) for all outcome variables. Overall impression of agreement depends on which measure used for the assessment. The previously reported desirable properties of Gwet’s AC1 as well as the patient characteristics associated with agreement were confirmed.

**Conclusion:**

The differences in incidence and agreement across the different variables and time points highlight the importance of carefully assessing which source of information to use in clinical research.

**Trial registration:**

ISRCTN06393679 (www.isrctn.com). Date of registration: 07/02/2008. Retrospectively registered.

## Background

In clinical research information can be retrieved through various sources. Three commonly used sources of information are the patient themselves (self-reported), professional caregivers (clinical reports) and medical records. The preferred source depends on the specific research objective and the kind of information retrieved from the different sources. However, there may be different sources of information to choose from for several types of data such as morbidity, symptoms and health care utilization [1].

Medical conditions are commonly assessed retrospectively through patient interviews or patient charts [2–7]. When assessing outcomes after cancer surgery, documenting patient’s comorbidity is crucial as it may influence outcomes [8], such as recurrence [9], symptoms, complications [10] and bodily dysfunctions [11]. Another aim is documentation of health care utilization [12, 13]. Whereas studies comparing self-reported questionnaires with medical records are common [3, 6, 14–17], evaluation of clinical reports has received less attention [2, 6, 8].

Knowledge of quality characteristics such as agreement and reliability of different sources of information is important. Evaluating agreement between different sources contributes to the choice of source for information in clinical research. The focus in this study is on the agreement between patient questionnaires and case report form in a study of patients after radical prostatectomy. Assessing agreement by a single measure is often insufficient [18]. The use of measures of agreement for continuous outcomes was explored in a systematic review [19]. For categorical outcomes where data is classified according to concordant (positive/positive or negative/negative) and discordant (positive/negative or negative/positive) pairs, any recent systematic review is to our knowledge currently not available. However, according to Wongpakaran [20] and by our current review of the literature, many studies only report Cohen’s Kappa [21], sometimes combined with positive and negative agreement [18]. Kappa is a measure of the level of agreement in excess of chance, expressed as the relative difference in proportions of concordant pairs between observed and what would have occurred by chance. Positive and negative agreement estimates the conditional probability that, given that one of randomly selected rater makes a positive and negative rating, respectively, the other rater will also do so. A limitation with kappa is its dependence on the underlying prevalence, giving rise to for example low kappa values despite high percentage of agreement [18, 22]. This originates from how chance agreement is computed [23]. Gwet [24] proposed a statistic, named AC1, which is similar to kappa but use an estimator of chance agreement that is less dependent on the prevalence [20]. In the correction for chance agreement of kappa, it is assumed that all observed ratings may potentially agree by pure chance. In Gwet’s AC1, the likelihood of chance agreement is instead related to the proportion of ratings that may lead to an agreement. This portion does in turn depend on the observed marginal prevalence’s. This enables Gwet’s AC1 to avoid the problem of over or under correction that Kappa suffer from.

Agreement can be analyzed by generalized linear models for contingency tables [25]. Log-linear models are a class of models which to enable to quantify the contribution of row, column and diagonal effects as well as covariates on the outcome. The quasi-independence model [26] enables a greater degree flexibility and a more in-depth study of the structure of agreement than simple summary measures. For the situation where a source provides the true conditions (positive/negative), referred to as gold standard, the sensitivity and specificity of the different sources can be evaluated. Sensitivity and specificity measures the rate of cases being correctly classified as positive and negative, respectively.

The aim of this study was to evaluate the agreement between patient questionnaires and clinical reports in a study of patients after radical prostatectomy and to study patient characteristics associated with agreement between these two data sources.

## Methods

Laparoscopic Prostatectomy Robot Open (LAPPRO) is an ongoing open, controlled and non-randomized prospective longitudinal trial comparing open retropubic and robot-assisted laparoscopic radical prostatectomy for localized prostate cancer at 14 centers in Sweden. The trial has been previously described in detail [27]. Patient scheduled for prostatectomy at the participating centers fulfilling the inclusion criteria (informed consent, age < 75 yr., ability to read and write Swedish, tumour stage cT1, cT2, or cT3 (TNM Classification of Malignant Tumors, [28]) with no signs of distant metastases, and a prostate-specific antigen level of < 20 ng/ml) were included.

**Fig. 1:**
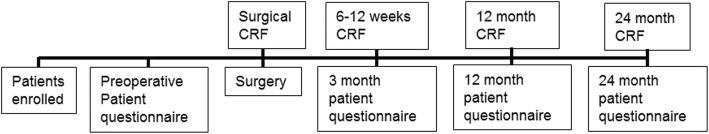
Flow chart of study procedures

Information was collected at 6–12 weeks, 12 and 24 months postoperatively both from case report forms by study personnel (either the operating urologist or clinical nurses) and from questionnaires filled out by the patients with regard to the following five outcomes: 1. swelling of groin or lower extremities, 2. complications and re-admissions, 3. re-operations, 4. added pharmacological therapy after surgery or due to local or distant recurrence and 5. local recurrence and metastases, see Fig. 1.

The questionnaires were based on concepts introduced in previous research projects [11, 29] and were content validated by experts in the field of urology and then face validated face with patients with prostate cancer. The case report forms were face validated with professional caregivers. The case report forms and questionnaires were further tested in a pilot study, after which final revisions were made [27]. The different questions in the case report forms and the questionnaires are presented in Supplement 1.

### Definition of outcomes and predictors

In the derivation of the outcome variables a broad approach was used with binary outcomes where the presence (positive) or absence (negative) of at least one occurrence of interest, for example a readmission, was required to be reported in both sources (case report form and questionnaire) to reach agreement. A higher level of similarity, for example the exact number of readmissions, was not required. The reason is that the recall of the patient and the case report form may not refer to exactly the same time period. The derived outcomes can hence be grouped according to concordant (positive/positive or negative/negative) and discordant (positive/negative or negative/positive) pairs.

When the recall of the patient and the case report form refer to the same time period a higher agreement is more likely compared to when there is no defined time period. However, in the study the personnel did to some extent complete the clinical record form retrospectively by investigating medical records. Therefore the time period cannot be accurately assed and is not used in the analysis. Lastly, the events studied here were anticipated to have relatively low incidence.

#### Swelling of groin or lower extremities

At 6–12 weeks follow-up the case report form addressed signs of swelling (lymph oedema) in groin (left or right side) and lower extremities (left or right) with response categories “*Yes*”/“*No*”. At 3 months the questionnaire addressed feeling of swelling and heaviness in (left or right) groin or leg and heaviness in legs. A positive outcome was defined as “*Yes”* on at least one of the questions. A negative outcome was defined as responding *No* on all the questions. Otherwise the outcome was set to missing.

#### Complications, readmissions and reoperations

The case report form collected information on complications related to surgery, complications occurring after 6–12 weeks, and if the patient had been re-admitted to hospital for other reasons than cancer treatment later than the 12 month follow-up. At 3, 12 and 24 months the questionnaire included a question on whether the patient had contacted healthcare for a specified list of reasons. If any reason included pain from the surgical wound, lower or upper part of abdomen, bleeding from surgical wound, urinary tract or catheter it was defined as an event. There was also a question on whether that patient had been readmitted to hospital. For the questionnaire, a positive outcome was defined as responding “*Yes”* on any of the questions. For the case report form positive and negative outcome was defined as responding “*Yes”* and “*No”*, respectively. Responding “*No information”* and a non-response outcome were defined as missing.

At 12 and 24 months the case report form addressed whether the patients had been re-operated after 6–12 weeks and 12 months follow-up, respectively. The questionnaire addressed if the patient had been operation during the last 12 months. For both the case report form and the questionnaire a positive and negative outcome was defined by responding “*Yes”* and “*No”*, respectively. A non-response outcome was defined as missing.

For a yet unpublished report, [30] data on all readmissions within 3 months of surgery were collected from the Patient registry, Swedish Board of Health and Welfare. These data will be compared with the questionnaire and the CRF at 3 months.

#### Adjuvant therapy and local recurrence and metastases

Signs of local recurrence and detection of distant metastases were assessed in the case report form at 12 and 24 months and at the same follow-up times the patients were also asked about these matters. For both the case report form and the questionnaire a positive and negative outcome was defined by responding “*Yes”* and “*No”*, respectively. For a non-response the outcome was defined as missing.

#### Predictor variables

Patient characteristics and demography were collected through the questionnaires preoperatively and throughout the study. Preoperatively, information on age, education, occupation and marital status was collected and evaluated with regard to association with agreement [7, 13, 16]. In addition, use of medication, alcohol consumption, quality of life, depressed mood and presence of negative intrusive thoughts were also evaluated. Use of medication was defined as use of sleeping pills or tranquilizers. Self-assessed quality of life, negative intrusive thoughts and alcohol consumption were characterized in the same way as in an earlier analysis [31]. Depressive mood was defined as either responding ‘Yes’ to the question ‘Would you call yourself depressed?’ [32] or use of anti-depressive medication.

#### Sensitivity and specificity

Sensitivity and specificity will be evaluated for two scenarios with regard to choice of gold standard. First, the questionnaire will be considered as gold standard and the sensitivity and specificity of the clinical reports will be evaluated. Secondly, the Patient registry will be considered as standard regarding readmissions and the questionnaire and clinical report will be evaluated.

### Statistical analysis

Group sizes in LAPPRO were set to evaluate urinary incontinence [27] and were judged to be sufficient to assess the current aim. Agreement was evaluated by percent of concordant pairs, positive and negative agreement and Gwet’s AC1. For comparison the kappa coefficient was computed as well. Association was evaluated by the odds that the two observers agree rather than disagree using the marginal quasi-independence model. Due to the hierarchical design where the surgeons are operating on several patients, who are longitudinally followed, there are dependency structures in the data that should ideally be accounted for in the statistical model. However, due to computational difficulties a standard fixed effect model was estimated separately at each time point.

In the evaluation of factors associated with agreement (positive or negative) between the two data sources, the following were evaluated: age, education, occupation, marital status, medication (sleeping pills or tranquilizers), alcohol consumption, quality of life, depressed mood and negative intrusive thoughts. For swelling of groin or lower extremities a standard simple logistic regression was used. For the outcomes with repeated measures a random intercept logistic regression model was used and time was included as a fixed effect ([26]). Results were presented with 95% confidence intervals. In each of the analyses, for information to be evaluable, data from both sources had to be ‘non-missing’ according to the definitions described above. The same analyses were made for the additional comparisons with data from the Patient registry. Analyses were conducted in SAS v9.4 (SAS Institute Inc., Cary NC), the *rel* package [33] and the software described by [34].

## Results

**Fig. 2:**
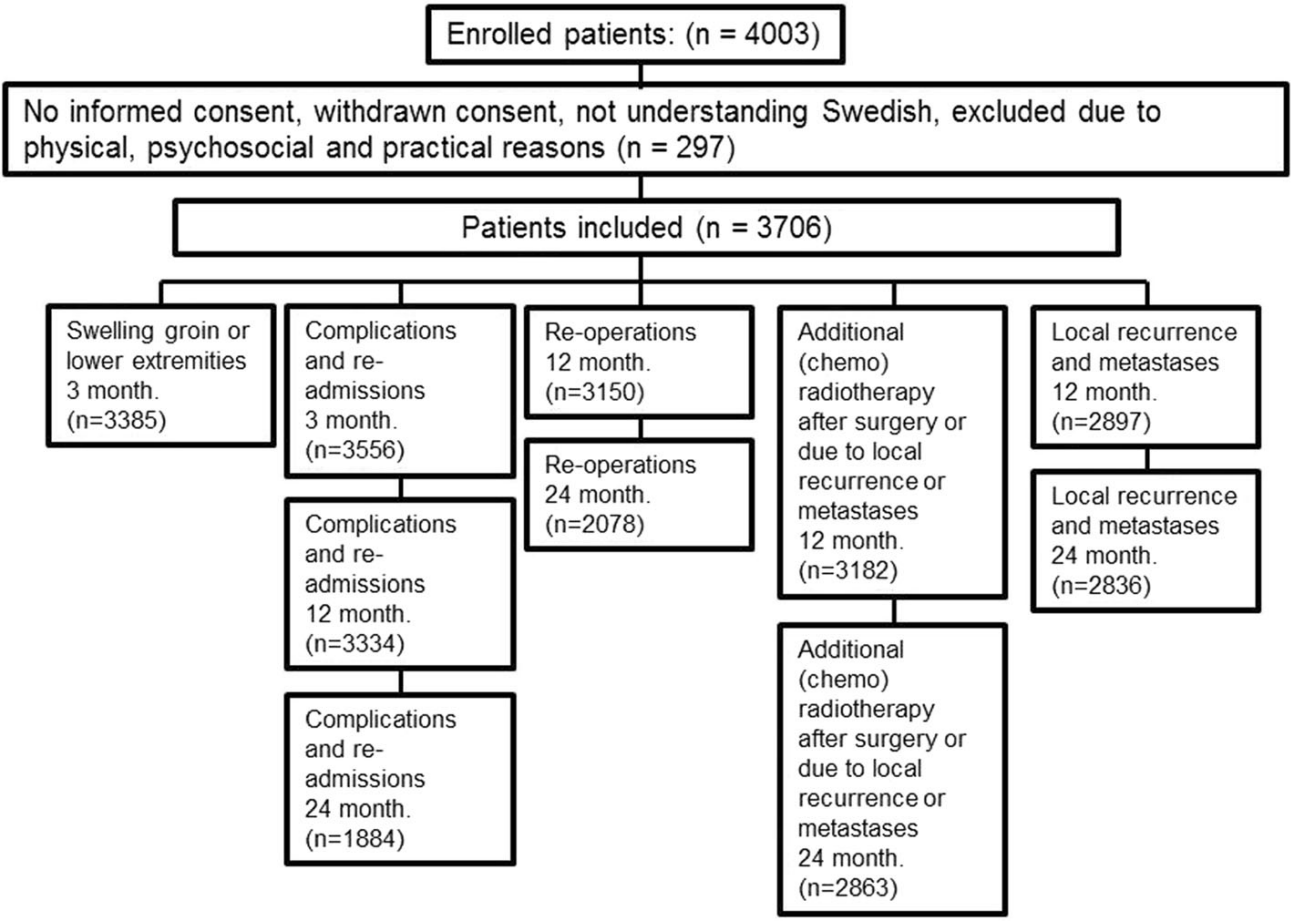
Flow chart of patients included in the study

For the 3706 eligible patients, the number of patients with evaluable data from both case report forms and questionnaires varied between the different questions from 3385 (91%) for swelling of groin and lower extremities at 3 months to 1884 (51%) for complications and readmissions at 24 months (Fig. 2). Missing information was consistently higher in the case report forms and increased at later follow-up (Table 1).

**Table 1:** Missing data

Assessment	Follow-up (months)	Missing data, N(%)		
		Case report form	Questionnaire	Missing on at least one
Swelling groin or lower extremities	3	163 (4%)	163 (4%)	321 (8%)
Complications and readmissions	3	150 (4%)	0	150 (4%)
	12	372 (10%)	0	372 (10%)
	24	1822 (49%)	0	1822 (49%)
Reoperations	12	300 (8%)	351 (9%)	556 (15%)
	24	1426 (38%)	356 (10%)	1628 (44%)
Additional (chemo) radiotherapy after surgery or due to local recurrence or metastases	12	251 (7%)	303 (8%)	524 (14%)
	24	555 (15%)	373 (10%)	843 (23%)
Local recurrence and metastases	12	809 (22%)	0	809 (22%)
	24	870 (23%)	0	870 (23%)

### Patient characteristics and demography

Patient characteristics are reported in Table 2. The median age of the patients was 63 years. Thirty-eight percent were retired and 84% were married/cohabiting.

**Table 2:** Patient characteristics

			Not Missing / Missing
Age, median (min; max)	Years	63 (37;79)	3706/.
Education, N (%)	No higher education	1948 (60)	3236/470
	Other	55 (2)	
	University	1233 (38)	
Occupation, N(%)	Working	1759 (54)	3257/449
	Retired	1253 (38)	
	Other	245 (8)	
Marital status, N (%)	Live apart	223 (7)	3235/471
	Married/cohabiting	2731 (84)	
	No partner	281 (9)	
Medication use ^a^	Yes	394 (12)	3253/453
Alcohol consumption, N (%)	High	427 (13)	3298/408
Global Quality of Life, N(%)	Low/moderate	1503 (46)	3234/467
Depression ^b^	Yes	265 (8)	3257/449
Negative intrusive thoughts	At least once per week	1170 (36)	3237/469

^a^Use of use of sleeping pills or tranquilizers

^b^Depressed mood or use of anti-depressants

### Agreement between case report forms and patient reported data

With the exception of local recurrence and metastases, all events were reported to a higher degree by the patient reports compared with the case report form (Table 3). The incidence of swelling of groin or lower extremities was 1 and 24% as reported by the case report form and questionnaire, respectively. Gwet’s AC1 was relatively stable and varied between 0.62 and 0.96 across outcomes and time points. Both kappa and the odds of agreement varied across a much wider range. Negative agreement was consistently higher than positive agreement for all the outcome variables.

**Table 3:** Evaluation of agreement between case report forms and patient reported data

Assessment	Follow-up (months)	No. (%)		Concordant pairs (%)	Agreement (95% CI)		Kappa (95% CI)	Gwet’s AC1 (95% CI)	Odds of agreement (95% CI)
		CRF^a^	Questionnaire		Positive	Negative			
Swelling groin or lower extremities	3	46 (1)	805 (24)	2620/3385 (77)	0.10 (0.07; 0.13)	0.87 (0.86; 0.88)	0.08 (0.06;0.10)	0.71 (0.69; 0.73)	48 (17.6;200.4)
Complications and readmissions^b^	3	373 (10)	1041 (29)	2624/3556 (74)	0.34 (0.31; 0.37)	0.84 (0.83; 0.85)	0.22 (0.19;0.25)	0.62 (0.59; 0.64)	5.4 (4.3;6.8)
	12	187 (6)	479 (14)	2884/3334 (87)	0.32 (0.28; 0.37)	0.93 (0.92; 0.93)	0.27 (0.22;0.31)	0.84 (0.82; 0.85)	10.2 (7.5;14)
	24	195 (10)	245 (13)	1554/1884 (82)	0.25 (0.20; 0.30)	0.90 (0.89; 0.91)	0.15 (0.09;0.21)	0.78 (0.75; 0.81)	3.1 (2.2;4.4)
Reoperations	12	115 (4)	348 (11)	2877/3150 (91)	0.41 (0.35; 0.47)	0.95 (0.95; 0.96)	0.38 (0.32;0.43)	0.90 (0.89; 0.91)	52 (32;88)
	24	171 (8)	264 (13)	1877/2078 (90)	0.54 (0.48; 0.60)	0.95 (0.94; 0.95)	0.49 (0.43;0.55)	0.88 (0.86; 0.90)	26 (18;38)
Additional (chemo) radiotherapy after surgery or due to local recurrence or metastases	12	212 (7)	260 (8)	3084/3182 (97)	0.79 (0.75; 0.83)	0.98 (0.98; 0.99)	0.78 (0.73;0.82)	0.96 (0.96; 0.97)	297 (187;489)
	24	207 (7)	233 (8)	2721/2863 (95)	0.68 (0.63; 0.73)	0.97 (0.97; 0.98)	0.65 (0.60;0.70)	0.94 (0.93; 0.95)	79 (55;115)
Local recurrence and metastases	12	159 (5)	49 (2)	2739/2897 (95)	0.24 (0.16; 0.32)	0.97 (0.97; 0.98)	0.22 (0.14;0.30)	0.94 (0.93; 0.95)	21 (12;38)
	24	197 (7)	44 (2)	2643/2836 (93)	0.20 (0.13; 0.27)	0.96 (0.96; 0.97)	0.18 (0.11;0.25)	0.93 (0.92; 0.94)	18 (10;34)

^a^ Case Report Form

^b^ Reasons: Pain in surgical wound, lower or upper part of abdomen, bleeding from surgical would, urinary tract or catheter

There was relatively high negative agreement across all variables and time points (84–97%) which rendered high odds of agreement. However, due to the low positive agreement for most of the variables and the low incidence as reported by the case report form, the kappa values were in general low. Gwet’s AC1 was less affected by the incidence. Agreement regarding additional (chemo) radiotherapy had higher agreement compared with the other variables.

Both reoperations and recurrence at 12 months had a relatively low incidence. However, despite being similar with regard to concordant pairs (91 and 95%, respectively) and negative agreement (95 and 97%, respectively), there was a large discrepancy in kappa (0.38 and 0.22) and odds of agreement (52 and 21) attributed to differences in incidence and positive agreement. Gwet’s AC1 were similar, 0.90 and 0.94, respectively. A similar pattern was observed at 24 months as well as for the comparison between additional (chemo) radiotherapy after surgery or due to recurrence at 12 and 24 months.

For the scenario where the questionnaire is regarded as gold standard the sensitivity of the case report form varied considerable between variables. Specificity was more stable at a high level which means that the case report forms have a higher likelihood of identifying absence rather than presence of events (Table 4).

**Table 4:** Evaluation of sensitivity and specificity of case report forms

Assessment	Follow-up (months)	Sensitivity (95% CI)	Specificity (95% CI)
Swelling groin or lower extremities	3	0.05 (0.04; 0.07)	0.99 (0.99; 1.00)
Complications and readmissions*	3	0.23 (0.21; 0.26)	0.95 (0.94; 0.96)
	12	0.23 (0.19; 0.26)	0.97 (0.97; 0.98)
	24	0.22 (0.17; 0.28)	0.91 (0.90; 0.93)
Reoperations	12	0.27 (0.23; 0.32)	0.99 (0.99; 1.00)
	24	0.44 (0.38; 0.50)	0.97 (0.96; 0.98)
Additional (chemo) radiotherapy after surgery or due to local recurrence or metastases	12	0.72 (0.66; 0.77)	0.99 (0.99; 0.99)
	24	0.64 (0.58; 0.70)	0.98 (0.97; 0.98)
Local recurrence and metastases	12	0.51 (0.37; 0.65)	0.95 (0.95; 0.96)
	24	0.55 (0.40; 0.69)	0.94 (0.93; 0.95)

* Reasons: Pain in surgical wound, lower or upper part of abdomen, bleeding from surgical would, urinary tract or catheter

### Agreement with patient registry data

In the comparisons of the questionnaire and CRF with Patient registry data, the estimated readmission rate was 1083 (29%), 373 (10%) and 291 (8%), respectively. The questionnaire had lower agreement with the registry compared with the CRF. The questionnaire had slightly higher sensitivity than the CRF. The CRF had higher specificity (Table 5).

**Table 5:** Re-admission within 3 months after surgery. Evaluation of agreement and sensitivity and specificity between patient registry and case report forms and patient reported data

Assessment	Concordant pairs (%)	Positive Agreement (95%)	Negative Agreement (95% CI)	Kappa (95% CI)	Gwet’s AC1 (95% CI)	Odds of agreement (95% CI)	Sensitivity (95% CI)	Specificity (95% CI)
Questionnaire	2716 (73)	0.28 (0.25; 0.31)	0.84 (0.83; 0.85)	0.18 (0.15; 0.21)	0.89 (0.88; 0.90)	5.5 (4.3; 7.1)	0.66 (0.60; 0.71)	0.74 (0.72; 0.75)
Case report forms	3228 (91)	0.50 (0.45; 0.55)	0.95 (0.94; 0.96)	0.45 (0.40; 0.50)	0.61 (0.58; 0.65)	20.2 (15.3; 26.6)	0.58 (0.52; 0.64)	0.94 (0.93; 0.95)

### Factors associated with agreement

Being retired, using sleeping pills and/or tranquilizers as well as reporting an impaired quality of life, depressed mood and negative intrusive thoughts were all associated with a lower agreement for several of the outcome variables (Table 6). Being married/cohabiting was associated with a higher degree of agreement for swelling of the groin. Age was associated with reoperations, (chemo) radiotherapy and local recurrence and metastases with high age yielding poor agreement. No association was found for education or alcohol consumption.

**Table 6:** Evaluation of patient characteristics associated with agreement between patient self-assessed questionnaires and case report forms

Variable	Comparison	Odds ratio (95% CI) ^a^				
		Swelling groin or lower extremities	Complications and readmissions^b^	Reoperations	Postoperative (chemo) radiotherapy, local recurrence or metastases	Local recurrence and metastases
Education	No higher education vs Other	1.30 (0.70; 2.42)	0.86 (0.54; 1.38)	0.71 (0.28; 1.78)	1.46 (0.58; 3.67)	0.80 (0.29; 2.22)
	No higher education vs University	0.92 (0.77; 1.10)	1.05 (0.93; 1.18)	0.92 (0.74; 1.13)	0.83 (0.61; 1.12)	1.13 (0.89; 1.43)
	Other vs University	0.71 (0.38; 1.33)	1.22 (0.76; 1.96)	1.29 (0.51; 3.25)	0.57 (0.22; 1.44)	1.40 (0.51; 3.90)
Occupation	Other vs Retired	0.78 (0.59; 1.09)	0.83 (0.67; 1.03)	1.70 (1.05; 2.73)	2.19 (1.05; 4.54)	1.23 (0.79; 1.91)
	Other vs Working	0.80 (0.58; 1.10)	0.78 (0.63; 0.96)	1.44 (0.90; 2.32)	1.57 (0.76; 3.25)	0.75 (0.48; 1.18)
	Retired vs Working	1.02 (0.85; 1.23)	0.94 (0.83; 1.06)	0.85 (0.69; 1.05)	0.72 (0.54; 0.96)	0.62 (0.49; 0.78)
Marital status	Live apart vs Married/cohabiting	0.68 (0.49; 0.93)	0.98 (0.78; 1.23)	0.90 (0.60; 1.34)	0.98 (0.56; 1.71)	1.20 (0.75; 1.94)
	Live apart vs No partner	0.79 (0.52; 1.19)	1.07 (0.80; 1.43)	0.89 (0.53; 1.50)	0.86 (0.41; 1.80)	0.72 (0.37; 1.39)
	Married/cohabiting vs No partner	1.17 (0.87; 1.57)	1.10 (0.90; 1.34)	0.99 (0.68; 1.44)	0.86 (0.51; 1.50)	0.60 (0.37; 0.97)
Medication	Medication vs No medication	0.76 (0.59; 0.97)	0.72 (0.61; 0.85)	0.80 (0.60; 1.06)	0.55 (0.38; 0.79)	0.95 (0.68; 1.33)
Alcohol consumption	Little alcohol vs Much alcohol	0.89 (0.69; 1.16)	1.29 (1.09; 1.51)	1.08 (0.81; 1.46)	0.95 (0.63; 1.43)	1.28 (0.94; 1.75)
Quality of Life	Low QoL vs High QoL	0.68 (0.57; 0.81)	0.73 (0.65; 0.82)	0.84 (0.69; 1.03)	0.86 (0.65; 1.13)	0.88 (0.70; 1.11)
Depressed mood	Depressed mood vs No depressed mood	0.66 (0.49; 0.88)	0.70 (0.58; 0.85)	1.31 (0.87; 1.98)	0.2 (0.55; 1.52)	1.06 (0.69; 1.61)
Negative intrusive thoughs	Intrusive thoughts vs No intrusive thoughts	0.65 (0.55; 0.78)	0.71 (0.63; 0.80)	0.93 (0.75; 1.14)	0.66 (0.50; 0.88)	0.69 (0.55; 0.87)
Age	Increase in age by 25 years	0.82 (0.59; 1.13)	0.86 (0.67; 1.10)	0.49 (0.32; 0.74)	0.32 (0.18; 0.58)	0.46 (0.28; 0.77)
Postoperative Time trend	24 months vs 12 months	Not applicable	0.74 (0.64; 1.15)	0.89 (0.74; 0.93)	0.61 (0.47; 1.26)	0.78 (0.63; 1.02)

^a^ Ratio of odds for agreement (positive or negative) between patients (questionnaire) and clinical report (case report form)

## Discussion

This study indicates that in a clinical trial, patients in general report a higher frequency of events than professional caregivers do. In the current study of patients after radical prostatectomy, with the exception of tumor recurrence, the incidence of various postoperative symptoms or events after radical prostatectomy for prostate cancer was consistently more frequently reported by the patients than in clinical reports. Missing information was consistently higher in the case report forms and increased at later follow-up.

Positive agreement was consistently lower than negative agreement for all the outcome variables. This is probably due to the relatively low incidence estimates and the large discrepancies in estimates between the patient and the clinical reports. Whereas both kappa and the odds of agreement varied across a wide range across the different variables and time points, despite other data characteristics of agreement being relatively similar, Gwet’s AC1 was relatively stable, which confirm previous findings [20, 24].

Accuracy and agreement between the different modes depends on what information is being collected [8]. For symptoms as well as events such as readmissions, an advantage with the questionnaire is that data is collected directly from the patient it concerns. However, the accuracy of recall may be questionable, especially in a retrospective setting with a long recall period [1]. Patients are more likely to recall a disease requiring a surgical or intensive pathologic/laboratory diagnostic procedure than a disease without such a procedures [14]. The time elapsed since the illness occurred and the seriousness of the disease influence agreement [3]. Diabetes has generally high agreement whereas chronic obstructive pulmonary disease and diseases with less explicit diagnostic criteria have poor agreement [6, 15]. For medical records recall bias is less of an issue as information is generally prospectively documented during a hospital admission that is data is recorded instantaneously in the course of time. A major limitation is that medical records may not cover the necessary information relevant for specific research objectives and underestimate symptoms based conditions [35]. They may also lack coverage as they miss patients not seeking health care or patients seeking health care in another county, or due to differences in reporting inpatient and outpatient visits [36]. An advantage with clinical reports is that they can be fit for purpose as opposed to medical records. A drawback is that information is filtered through the physician [11]. In the collection of signs, different types of medical examinations by an experienced clinician may be the only viable option.

The patient may be less prone to report complications when the clinical personnel asks for information compared with when he or she completes an anonymous questionnaire at home [37]. In this trial questionnaires were sent out and returned to a third party, the trial secretariat and not to the hospital/department where surgery was performed [27]. The information documented in the clinical record form was collected when clinical personnel met the patient during the follow-up meetings as well as from medical records. This probably explains the observed high agreement between the clinical record forms and the patient registry. A higher agreement between patient reports and medical records compared with patient reports versus physician reports has also been observed [8].

It has been found that patients with prostate cancer reported a higher incidence of symptoms such as fatigue compared to their physicians [38, 39]. A similar pattern was found in [40] but with a higher degree of agreement. Several study design features may contribute to these differences as discussed in [40].

Several of the studies ([5, 6, 9, 14, 16]) have used medical records as the gold standard enabling assessment of sensitivity and specificity. However, it may be an invalid assumption for other settings [35]. For the scenario where the Patient registry was regarded as the gold standard in reporting readmissions, the case report forms had a higher agreement and specificity compared to the questionnaire, whereas the questionnaire had a slightly higher sensitivity.

In our study some patient characteristics were found to be associated with agreement such as age, socioeconomic factors and depressed mood, which confirmed previous results [7, 13, 16]. Younger patients, who had no self-reported impairment in quality of life, depressed mood or negative intrusive thoughts, appear to report symptoms events more in agreement with those reported by clinical personnel. However, the association with medication (sleeping pills and/or tranquilizers) has not been previously reported as far as we know.

At later follow ups (12 and 24 months) the compliance in submitting clinical record forms was significantly lower compared with compliance regarding patient reports. One reason for this could be that part of the cohort was referred for surgery to a department of urology some, or even a long, distance from their home. This would be expected to result in a lower surgeon-patient physical follow-up (out-patients visit). Other contributing explanations could be that after 12 or 24 months the follow-up was not always by the operating urologist, and thus completion of the CRF may have been missed.

This study has both strengths and limitations. Strengths include the large study cohort, the longitudinal design, a high compliance of patients and the use of validated questionnaires [27]. Limitations include a lack of information of the specific personnel who completed the case report forms at the different visits and on the specific time periods the case report forms and questionnaires covered. Difficulties in being able to account for the longitudinal structure in the statistical model must be regarded as a limitation.

## Conclusions

The differences in incidence and agreement across the different variables and time points highlight the importance of carefully assessing which source of information to use in clinical research. This study confirms the importance of using several measures to assess the degree of agreement between the sources. The previously reported benefits of Gwet’s AC1 are confirmed and researchers should be encouraged to consider this method. In clinical research, much effort is often devoted to increasing patient response rates. However, preventing missing data in clinical reports also needs further attention. Long-term follow-up should make use of patient reports, as clinical record forms tend to be missed by the health care. As different patient characteristics were found to increase agreement, such background information is relevant for the choice of data collection procedure.

## Abbreviations

CRF: Case Report Form
LAPPRO: Laparoscopic Prostatectomy Robot Open trial
